# Effect of Dietary Energy Level during Late Gestation on Mineral Contents in Colostrum, Milk, and Plasma of Lactating Jennies

**DOI:** 10.3390/ani14162383

**Published:** 2024-08-16

**Authors:** Fang Hui, Manman Tong, Shuyi Li, Yanli Zhao, Xiaoyu Guo, Yongmei Guo, Binlin Shi, Sumei Yan

**Affiliations:** Inner Mongolia Key Laboratory of Animal Nutrition and Feed Science, College of Animal Science, Inner Mongolia Agricultural University, Hohhot 010018, China; hf2021@emails.imau.edu.cn (F.H.); tongmm@emails.imau.edu.cn (M.T.); lsy2835245597@emails.imau.edu.cn (S.L.); ylzhao2017@imau.edu.cn (Y.Z.); gxy2024@imau.edu.cn (X.G.); ymguo2020@imau.edu.cn (Y.G.); shibl@imau.edu.cn (B.S.)

**Keywords:** jennies, colostrum, milk mineral, dietary energy, pregnancy

## Abstract

**Simple Summary:**

Donkey milk is recognized as a functional food due to its high whey protein content. It is especially beneficial for newborn nutrition because of its nutritional similarities to human milk and its hypoallergenic properties. It can be used to prevent hypercholesterolemia and atherosclerosis. However, donkey lactation is less productive in terms of liters/d than dairy cow lactation. It has been suggested that the energy content of the diet in late pregnancy is the main factor influencing the composition of postpartum colostrum in dairy animals. However, research on the influence of dietary energy in late gestation on the mineral content of postpartum jenny milk is limited. Therefore, this study aimed to investigate the effect of dietary energy levels during late gestation on mineral contents in the colostrum milk of lactating jennies. The results showed that appropriately increasing dietary energy levels in late gestation increased the concentrations of Ca, P, K, Mg, Cu, Fe, Zn, and Mo in milk, but high dietary energy levels showed the opposite effect. The concentrations of these minerals in jenny milk decreased with the duration of lactation.

**Abstract:**

This study investigated the effects of dietary energy levels during late gestation on mineral content in the plasma, colostrum, and milk of jennies postpartum. Twenty-four pregnant multiparous DeZhou jennies, aged 6.0  ±  0.1 years, with a body weight of 292  ±  33 kg, an average parity number of 2.7  ±  0.1, and similar expected dates of confinement (74  ±  4 days), were randomly allocated to three groups and fed three diets: high energy (12.54 MJ/kg, HE), medium energy (12.03 MJ/kg, ME), and low energy (11.39 MJ/kg, LE). Blood samples were collected from the jugular vein of each jenny at time points of 0 h, 24 h, 48 h, 5 d, 7 d, and 14 d after parturition. Additionally, milk samples were collected through manual milking, and an analysis of the mineral content was conducted. The results showed that compared with HE, both ME and LE significantly increased the levels of calcium (Ca), phosphorus (P), zinc (Zn), selenium (Se), molybdenum (Mo), and cobalt (Co) in the plasma and Ca, P, magnesium (Mg), copper (Cu), manganese (Mn), Zn, selenium (Se), molybdenum (Mo), and Co in the milk of jennies postpartum (*p* < 0.05); ME also increased the levels of potassium (K), iron (Fe), and Mn in plasma and K and Fe in milk (*p* < 0.05). The levels of Ca, K, Mg, P, Fe, Cu, Mn, Co, Se, Zn, and Mo in plasma and milk gradually decreased with increasing postpartum time. Their contents were the highest at 0 h postpartum, rapidly decreased after 24 h postpartum, and declined to the lowest on day 14 postpartum. The interaction between dietary energy level and postpartum time showed that although the concentrations of the minerals Ca, P, K, Mg, Fe, Cu, Mn, Zn, Co, Se, and Mo decreased in jennies’ plasma and milk in the treatment groups with different energy levels as postpartum time increased, the pattern of change was also influenced by dietary energy level. The influence of dietary energy level in late gestation on the mineral content of milk and plasma during the postpartum colostrum phase was higher than that during the milk phase. In conclusion, this study demonstrated that, under the current experimental conditions, the mineral content of the colostrum, milk, and plasma of jennies after parturition was dependent on the dietary energy level during late gestation.

## 1. Introduction

Colostrum is the first food of newborn foals, with well-known properties related to high energy content (fat content) and protein (maternal immunoglobulin provides passive but temporary immunity). Mineral elements, as one of the critical components of jennies’ milk, are not only the main route of mineral intake for jennies’ foals after birth, but also play an essential role in their immune-antioxidant function. Blood mineral concentration is an important indicator of the mineral nutritional status of an animal and is closely related to the mineral concentration in milk [1]. The stage of lactation is a significant factor influencing blood and milk mineral concentrations. Minimal studies have shown that the concentration of mineral elements in jennies’ milk exhibits a decreasing trend with the duration of lactation [2], and Hui et al. [3] indicated that concentrations of various minerals in serum showed a similar pattern of change with the duration of lactation. Hu et al. [4] and Long et al. [5] obtained similar results for sows’ milk. The content and proportion of mineral elements in colostrum differed from those of milk, reflecting the characteristics of mammary tissue in mineral metabolism at different stages of lactation. In addition, it was reported that low-energy feeding during the periparturient period increases postpartum milk production in dairy cows [6]. It has also been reported that excessive energy intake in sows during gestation reduced feed intake during lactation [7] and negatively affected milk production [8]. However, Fang et al. [9] reported that sows fed diets with a metabolizable energy of 13.40 or 13.82 MJ/kg during gestation had improved reproductive performance, farrowing performance, and colostrum quality with dietary additions of 13.82 MJ/kg metabolizable energy. Heo et al. [10] assigned sows to diets with increasing energy density during late gestation and lactation (13.1, 13.4, and 13.7 MJ DE/kg), finding that fat and lactose content in sow colostrum increased with higher dietary energy density. Similarly, Che et al. [11] reported comparable results. These results suggested that the energy level of diets in late gestation appears to be among the conditioning factors affecting colostrum composition; however, studies have reported inconsistent results, and research in this area remains limited. Although our previous study found that dietary energy levels in late-pregnant jennies significantly affected their antioxidant capacity, immune function, nutrient digestion, and metabolism prepartum [12], there remains a lack of data regarding the impact of dietary energy levels during late gestation on mineral content in milk after parturition. Therefore, this study aimed to investigate the effects of feeding diets with varying energy levels during late gestation on the mineral content of colostrum, milk, and plasma in lactating jennies after parturition.

We hypothesized that feeding diets with varying energy levels during late gestation would significantly affect plasma and milk mineral concentrations in jennies. Additionally, we anticipated that increased energy intake during lactation would result in higher mineral concentrations in colostrum and milk.

Given this, this experiment was conducted to investigate the effects of feeding diets with different energy levels during the late gestation period on the mineral content of the colostrum, milk, and plasma of jennies after delivery to provide data support and a theoretical basis for the supply of appropriate amounts of energy to pregnant jennies and the optimal production of jennies’ milk, and for understanding the characteristics of the minerals in jennies’ milk.

## 2. Materials and Methods

This experiment was conducted in the Research Station of Inner Mongolia Agricultural University (Hohhot, China). The animal experimental procedures were performed in accordance with the National Standard Guidelines for Ethical Review of Animal Welfare (GB/T 35892-2018).

### 2.1. Experimental Design, Diet, and Feeding Management

Twenty-four pregnant multiparous DeZhou jennies, aged 6.0  ±  0.1 years, with a body weight of 292  ±  34 kg, an average parity number of 2.7  ±  0.1, and similar expected dates of confinement (74  ±  4 days), were randomly allocated to three groups and fed three diets: high energy (12.54 MJ/kg, HE), medium energy (12.03 MJ/kg, ME), and low energy (11.39 MJ/kg, LE). The ingredients and nutrient compositions of the diets are shown in Table 1. The experiment lasted 88 days: the initial 14 days were for the pretrial period, and in the following 60 days, the groups were fed their respective diets, and the same lactation diet was fed uniformly for 14 days after foaling. The jennies were kept in individual pens and fed twice daily at 07:30 and 14:00, respectively. The concentrate-to-forage ratio of the ration was kept constant at 30 to 70. The jennies had free access to the diets and water.

### 2.2. Measurements and Collection

Blood was collected from the jugular vein of each jenny at 0 h, 24 h, 48 h, 5 d, 7 d, and 14 d after parturition, and milk samples were collected by manual milking of the lactating jennies twice daily, at 10:00 h and 17:00 h. Foals were physically separated from the dams 3 h before milking, and eye contact with and visibility of the jennies were maintained. The milk samples from the morning and afternoon sessions were mixed in equal proportions, with a final volume of no less than 200 mL per jenny, dispensed into several 50 mL centrifuge tubes, and frozen at −20 °C until analyzed. The macronutrient and trace element contents of the plasma and milk of the jennies at different times were determined by wet digestion [14], using an inductively coupled plasma-emission spectrometer (ICP-OES) on an ICAP 6300 Duo spectrometer (Thermo Fisher, Waltham, Massachusetts, USA). The macronutrients included calcium (Ca), phosphorus (P), potassium (K), and magnesium (Mg), and the trace elements included iron (Fe), copper (Cu), manganese (Mn), zinc (Zn), cobalt (Co), selenium (Se), and molybdenum (Mo). The specific determination procedure was the Fantuz et al. [15] method. Briefly, 1 mL of the sample was placed in a Teflon digestion vessel, followed by adding 3 mL of HNO3 (65%, Suprapur quality, Merck, Darmstadt, Hesse, Germany). A microwave closed-vessel system was employed for digestion. The digested solutions were then transferred to a 10 mL volumetric flask and diluted with ultrapure water to achieve a specific concentration for elemental analysis using appropriate analytical instrumentation.

### 2.3. Statistical Analysis

The mean calculated for the data in this trial was the least-squares mean, and statistical analyses were performed in SAS software (version 8.1, SAS Institute, Cary, NC, USA). The treatment effects of the minerals in plasma and milk were analyzed using the PROC MIXED procedure as follows:

Y_ijk_ = μ + L_i_ + E_j_ + LE_ij_ + ε_ijk_,

where Y_ijk_ is the dependent variable; μ is the overall mean; L_i_ is the fixed effect of different energy levels of the ratio (i = 1 to 3); E_j_ is the fixed effect of different times postpartum (j = 1 to 6 for 0 h, 24 h, 48 h, 5 d, 7 d, and 14 d, respectively); LE_ij_ is also considered a fixed effect of the interaction of different energy levels of the ration with different times postpartum; and ε_ijk_ is the residual error. Pearson’s method was used to analyze the correlation between the elements in jennies’ plasma and milk. Differences with *p* < 0.05 were considered significant, differences with 0.05 ≤ *p* ≤ 0.10 were considered to indicate a tendency toward statistical significance, and adjusted *p*-values were used in the PROC MIXED procedure.

## 3. Results

### 3.1. Plasma Mineral Content

The plasma Ca and P concentrations in group ME were significantly higher than those in groups HE and LE (*p* < 0.05), and in group LE, they were significantly higher than in group HE (*p* < 0.05); the plasma K concentrations in group ME were significantly higher than those in groups HE and LE (*p* = 0.001), and there was no difference among groups HE and LE (*p* > 0.05). There was no significant effect on plasma Mg contents among the three groups during the total experimental period (*p* > 0.05) (Table 2).

Postpartum time had a significant effect on plasma Ca, P, K, and Mg levels (Table 2), which decreased with increasing postpartum time, and the values within 5 days were significantly higher than on days 7 and 14 (except for 7 d postpartum in the case of Mg) (*p* < 0.05). They were significantly higher at 0 h, decreasing to a minimum on day 7 or 14.

There were significant interactions among the DE and PT and Ca, P, K, and Mg plasma levels (*p* < 0.001) (Table 3). Ca content was highest in groups ME and LE at 0 h, significantly higher than the other combinations (*p* < 0.05), but lower on day 14 in groups HE and LE. P content was highest at 0 h in group ME, significantly higher than the other combinations (*p* < 0.05), but lower on days 7 and 14 in all three groups. K content was higher at 0 h in the HE, ME, and LE groups, being significantly higher at 0 h postpartum in group ME, compared to the other combinations (*p* < 0.05), but lower on days 7 and 14 in all three groups. Mg content was higher in groups HE and LE at 0 h, at 0 h, and 24 h in group ME, with the highest content found in group ME at 0 h, which was significantly higher than the other combinations (*p* < 0.05), but lower at 48 h–14 d in all three groups.

Plasma Fe content was significantly higher in group ME than groups HE and LE (*p* = 0.001), while the difference among the remaining two groups was not significant (*p* > 0.05); plasma Mn content was significantly higher in group ME than in group HE (*p* = 0.003), but the difference among LE and the rest of the two groups was not significant (*p* > 0.05); plasma Zn content in group ME was significantly higher than that in groups HE and LE, and that in group LE was significantly higher than that in group HE (*p* < 0.001); plasma Se, Mo, and Co contents were significantly higher in groups ME and LE than in group HE (*p* < 0.05), whereas the difference among the LE and ME groups was not significant (*p* > 0.05). There was no significant effect on plasma Cu contents among the three groups during the whole experimental period (*p* > 0.05) (Table 4).

Postpartum time had a significant effect on plasma Fe, Cu, Mn, Zn, Se, Mo, and Co levels (*p* < 0.05), and all of them decreased with postpartum time; among them, Fe, Mn, Zn, Se, and Co levels were highest at 0 h, significantly higher than at the other time points (*p* < 0.05); all of them decreased significantly starting at 24 h, and then reduced to the lowest at 14 d. Cu and Mo levels were higher at 24 h, significantly higher than at the other time points (except for Cu levels at 48 h) (*p* < 0.05), and decreased to the lowest on day 14 (Table 4).

There were significant interactions among DE and PT and Fe, Cu, Mn, Zn, Se, Mo, and Co plasma levels (*p* < 0.001) (Table 5); among them, Fe was higher in groups ME and LE at 0 h, and was significantly higher at 0 h in group ME, compared to the other combinations (*p* < 0.05); Cu was higher at 0 h–48 h in groups HE and LE and 0 h–5 d in group ME, and was significantly higher at 0 h in group ME, compared to the other combinations (*p* < 0.05), but lower on days 7 and 14 in group HE and days 5–14 in group LE. Mn was higher at 0 h in group HE and at 24 h in groups ME and LE, and second highest at 24 h in group HE, especially at 0 h in groups ME and LE, compared to the other combinations (*p* < 0.05), and lower at 0 h in groups HE and LE. Zn content was highest at 0 h in group ME, significantly higher than the other combinations (*p* < 0.05), but lower on days 7 and 14 in group HE and 14 in group LE. Se was higher at 0 h in group HE and at 24 h in groups ME and LE, especially at 0 h in groups ME and LE, but lower on day 14 in group HE. Mo was higher at 24 h in group HE, within 48 h in the ME group, and at 24 h in the LE group, and was highest at 0 h in the ME and LE groups in particular, compared to the other combinations (*p* < 0.05), but lower on day 14 in group HE. Co was higher at 0 h in group HE and 24 h in groups ME and LE, and highest at 0 h in groups ME and LE, compared to the other combinations (*p* < 0.05) but lower on day 14 in group HE.

### 3.2. Milk Mineral Content

Milk Ca, P, and Mg concentrations in group ME were significantly higher than those in groups HE and LE (*p* < 0.05), and those in group LE were significantly higher than group HE (*p* < 0.05); milk K content was significantly higher in group ME than that in groups HE and LE (*p* < 0.001), and there was no difference among groups HE and LE (*p* > 0.05) (Table 6).

Postpartum time had a significant effect on milk Ca, P, K, and Mg contents, and all of them decreased with postpartum time. In particular, milk Ca, P, K, and Mg contents were greater within 5 days, significantly higher than on days 7 and 14 (*p* < 0.05), and especially highest at 0 h postpartum, decreasing to a minimum on day 14 (Table 6).

There were significant interactions among milk DE and PT and Ca, P, K, and Mg levels (*p* < 0.001) (Table 7). Ca content was highest at 0 h in group ME, significantly higher than the other combinations (*p* < 0.05), but lower on day 14 in group HE. P content was highest at 0 h in group ME, significantly higher than the other combinations (*p* < 0.05), followed by 24 h in group ME and 0 h and 24 h in group LE, but lower on day 14 in groups HE and LE. K content was highest at 0 h in group ME, significantly higher than the other combinations (*p* < 0.05), but lower on day 14 in groups HE and LE. Mg content was highest at 0 h in group ME, significantly higher than the other combinations (*p* < 0.05), but lower on days 7 and 14 in groups HE and LE.

Milk Fe content was significantly higher in group ME than in groups HE and LE (*p* < 0.05), and there was no difference among groups HE and LE (*p* > 0.05); milk Cu, Zn, and Mo concentrations in group ME were significantly higher than those in groups HE and LE (*p* < 0.05), and those in group LE were significantly higher than those in group HE (*p* < 0.05); milk Mn, Se, and Co concentrations in group ME and LE were significantly higher than those in group HE (*p* < 0.05), and there was no difference among groups ME and LE (*p* > 0.05) (Table 8).

Postpartum time had a significant effect on milk Fe, Cu, Mn, Mo, Se, Zn, and Co content, which decreased significantly with postpartum time, and all were highest at 0 h postpartum, significantly higher than at the other time points (*p* < 0.05), and then began to decrease rapidly at 24 h, and then decreased to a minimum on day 14 (Table 8).

There were significant interactions among milk DE and PT and Fe, Cu, Mn, Zn, Se, Mo, and Co levels (*p* < 0.001) (Table 9), where Fe was highest at 0 h in the ME group, significantly higher than the other combinations (*p* < 0.05), but lower on day 14 in groups HE and LE. Cu content was highest at 0 h in group ME, significantly higher than other combinations (*p* < 0.05), but lower on day 14 in groups HE and LE. Mn was higher at 0 h in group ME, significantly higher than other combinations (*p* < 0.05), but lower on day 14 in group HE. Zn was highest at 0 h in group ME, significantly higher than other combinations (*p* < 0.05), but lower on days 7 and 14 in group HE and 14 d in group LE. Se content was higher at 0 h in groups ME and LE, significantly higher than the other combinations (*p* < 0.05), but lower on days 7 and 14 in group HE and day 14 in group LE. Mo content was higher at 0 h in groups ME and LE, significantly higher than the other combinations (*p* < 0.05), but lower on day 14 in all three groups. Co levels were higher at 0 h in groups ME and LE, significantly higher than in the other combinations (*p* < 0.05), but lower on day 14 in all three groups.

### 3.3. Correlation Analysis of Plasma and Milk Mineral Content in Jennies

Table 10 shows a significant positive correlation (*p* < 0.05) among plasma Ca, P, and K and Mg content and their content in milk.

Table 11 shows a significant positive correlation (*p* < 0.05) between plasma levels of Fe, Cu, Mn, Mo, Se, Zn, and Co and their respective levels in milk.

### 3.4. Average Content and Distribution Range of Minerals in Milk at Different Times after Parturition of Jennies

The results in Table 11 show that the distribution of Ca, P, K, Mg, Fe, Cu, Mn, Zn, Se, Mo, and Co in milk within 7 days after postpartum were in the ranges of 1043.7–1351.11 mg/L, 771.57–1218.31 mg/L, 465.73–1539.78 mg/L, 78.99–472.26 mg/L, 156.87–278.47 μg/100 mL, 10.32–22.11 μg/100 mL, 2.16–4.71 μg/100 mL, 99.26–270.49 μg/100 mL, 0.63–2.44 μg/100 mL, 0.48–1.76 μg/100 mL, and 0.15–0.32 μg/100 mL, and were 1.31, 1.56, 2.56, 2.74, 1.62, 1.85, 2.03, 2.07, 3.28, 2.92, and 1.62 times higher than those 14 days postpartum, in that order. In particular, their content was higher from 0 h to 24 h postpartum, and their contents were 1232.54–1351.11 mg/L, 1114.91–1218.31 mg/L, 1278.12–1539.78 mg/L, 149.60–472.26 mg/L, 232.04–278.47 μg/100 mL, 16.22–22.11 μg/100 mL, 3.51–4.71 μg/100 mL, 199.35–270.49 μg/100 mL, 1.53–2.44 μg/100 mL, and 1.53–2.44 μg/100 mL, 2.44 μg/100 mL, 1.28–1.76 μg/100 mL, and 0.21–0.32 μg/100 mL were 1.45, 1.80, 3.92, 4.72, 1.98, 2.32, 2.60, 2.78, 4.96, 4.61, and 2.04 times higher than those 14 days postpartum, in that order.

## 4. Discussion

### 4.1. Effect of Dietary Energy Levels in Late Gestation on Mineral Content of Postpartum Colostrum and Milk of Jennies

Mineral elements play an important role in the life activities of the animal body. Colostrum is the first food of newborn foals, and a high mineral content is essential to ensure the foal’s intake of an adequate mineral amount. The energy level of the diet in the late stages of pregnancy is the main factor influencing the composition of colostrum and standing milk in the mother animal’s post-partum period, but many studies have focused on mammals such as cows and sows [6,9,16], and very few studies targeting the mineral elements of animal milk are available.

The current research concluded that different dietary energy levels during the late gestation period significantly affected the mineral concentrations in the blood and milk of jennies after parturition. The plasma concentrations of Ca, P, K, Fe, and Zn, as well as the milk concentrations of Ca, P, K, Mg, Cu, Fe, Zn, and Mo, were higher when jennies were fed the ME group diet than when they were fed the LE group diet. However, the high dietary energy did not further increase the Ca, P, K, Fe, and Zn contents in the plasma and milk of lactating jennies, and even reduced the concentration of these mineral elements. The study’s results also concluded that dietary energy’s effect on postpartum blood and milk mineral content was related to the duration of postpartum lactation. Specifically, plasma and milk mineral content were more affected by dietary energy levels during late gestation in the colostrum period, which lasts up to 7 days postpartum, and less so during the standing lactation period. Hare et al. [17] found that late gestation metabolizable energy intake increased colostrum yield and altered colostrum composition in beef cattle. Additionally, sows with excessive energy intake during gestation may reduce their lactation feed intake [7], negatively affecting milk production [8]. Conversely, it has been reported that sows fed diets with metabolizable energy levels of 13.40 or 13.82 MJ/kg during gestation can benefit from dietary additions of 13.82 MJ/kg metabolizable energy, improving reproductive performance, farrowing outcomes, and colostrum quality [9]. Heo et al. [10] assigned sows to diets with increasing energy densities during late gestation and lactation (13.1, 13.4, and 13.7 MJ DE/kg), finding that fat and lactose content in sow colostrum increased with higher dietary energy density. Similar results were reported in the study by Che et al. [11]. In contrast, other studies have indicated that peri-parturient low-energy feeding can increase postpartum milk production in dairy cows [6]. These findings suggest that the energy level of the diet during late gestation is a significant factor influencing the composition of postpartum colostrum in dairy animals. However, the results reported in related studies are inconsistent, indicating the potential for improving jennies’ milk composition by adjusting the diet’s energy level during late gestation. Therefore, this also suggests that the elevated mineral concentrations in the postpartum colostrum of jennies fed low- to medium-energy diets before parturition, as indicated by the present study, may be attributed to the feeding of diets with ap-propriate energy levels, which facilitates the absorption of minerals and enhances their effects.

In addition, the milk secreted by dairy animals in the early stage of lactation is limited and coarse, and the water content of the milk is low [18]. With the prolongation of the lactation period, the demand for breast milk from young jennies increases rapidly, the amount of milk and water secreted by jennies increases, and the content of each element appears to decline more significantly. The mineral content of breast milk is influenced by the uptake and absorption by the mammary gland, in addition to the concentration of minerals in the plasma. Minerals are acquired through active transport by the mammary epithelial cells themselves, which, in turn, allows for the aggregation of certain mineral elements in the milk and promotes the growth and development of the infant [19]. Thus, this also further explains the inconsistent effects of medium- and low-energy-level rations on colostrum and milk during late gestation, which needs to be explored in the future. It is known that the bioavailability of mineral elements varies according to their distribution among milk fractions, as well as the presence of other compounds such as ascorbate, citrate, lactose, or casein phosphopeptides. Therefore, it is necessary to explore the influence of dietary energy levels during late gestation on postpartum milk composition, including milk minerals.

### 4.2. Changes in Mineral Content of Colostrum Phase and Milk Phase

Species-specific colostrum traits and the impact of colostrum components on neonatal development and health have been the subjects of numerous scientific papers and reviews [20,21]. The first milk secreted after parturition (colostrum) is crucial for certain species to receive passive immunization with immunoglobulins (Ig). In equids, immunoglobulins (Igs) do not cross the placental barrier, and foals must absorb Igs through colostrum to gain immediate immune protection against environmental hazards [22]. In addition, colostrum is extremely high in Mg, P, Fe, and Ca, which together determine the diarrhea-retarding properties of colostrum. These substances, once ingested by young animals, can be absorbed into the bloodstream by the intestinal wall without intestinal decomposition, increasing plasma protein [23].

In different mammals, the mineral content of colostrum and regular milk varies considerably. Lu et al. [24] showed that the contents of Ca, P, Na, and Mg in the colostrum of dairy cows at 2 h postpartum were 1.90, 2.24, 2.11, and 5.56 times greater than those of milk, and the contents of Zn, Fe, and Mn were 6.21, 2.81, and 1.64 times greater than those of normal milk. Fantuz et al. [2] found that the concentrations of Ca and P in jennies’ milk were closely correlated to the lactation period and showed a decreasing trend during lactation. Zs et al. [25] studied the pattern of change in macro trace element content in mare colostrum and milk and pointed out that except for Ca, all trace elements (Zn, Fe, Cu, Mn) showed a decreasing trend in colostrum and early lactation. Summer et al. [26] also noted that the concentrations of Ca, P, and Mg in horses’ milk decreased as lactation progressed. The present study concluded that Ca, P, K, Mg, Fe, Cu, Mn, Zn, Co, Se, and Mo concentration changes in jennies’ milk followed a similar pattern. These mineral concentrations gradually decreased with the prolongation of the postpartum period and were highest at 0 h postpartum, and then rapidly decreased after 24 h postpartum, and mostly stabilized on day 7 postpartum.

Reports on blood mineral content in jennies are quite limited. Liao et al. [27] measured serum mineral levels in jennies during early lactation and found that the concentrations of serum Ca, P, K, and Mg were in the ranges of 111.20–142.00 mg/L, 29.14–40.61 mg/L, 145.47–190.32 mg/L, and 20.64–63.60 mg/L, respectively. Turke et al. [28] assessed trace elements in jennies’ serum in the eastern region of the Kingdom of Saudi Arabia and reported a wide range of serum Fe, Cu, Mn, Zn, and Se concentrations. Specifically, the distribution of these trace elements in serum was found to be 220–834 µg/100 mL for Fe, 106–312 µg/100 mL for Cu, 36–548 µg/100 mL for Mn, 6.4–12.6 µg/100 mL for Zn, and 3042–3512 µg/100 mL for Se. Furthermore, Fantuz et al. (2015) [15] reported a serum Mo level of 2.85 µg/100 mL in lactating jennies, while Fantuz et al. (2013) [29] found that the serum Co level was 0.10 µg/100 mL in the same population. In the present study, we found that the measured plasma Mg levels were within the reference range, while Fe levels were slightly elevated compared to the reference values. Conversely, the levels of the other elements did not completely align with the reference values. The inconsistency of these results may be attributed to variations in breed, diet, or the stage of lactation. Jennies have distinct mineral requirements at different physiological stages, influenced by their unique metabolic characteristics.

**Figure 1:**
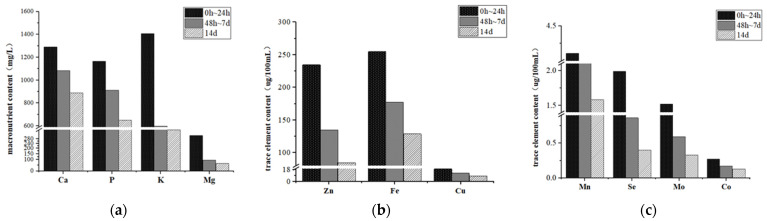
Trends in the mineral content of the milk of jennies at different times after parturition. (**a**) Trends in Ca, P, K, and Mg in the milk of jennies; (**b**) trends in Zn, Fe, and Cu in the milk of jennies; (**c**) trends in Mn, Se, Mo, and Co in the milk of jennies.

The present study also found the average content and range of distribution of minerals in milk from 0 h to 24 h, from 48 h to 7 days, and 14 days postpartum (Table 12 and Figure 1). These results suggested that colostrum, which is rich in minerals, is an important source of mineral supplementation to guarantee that foals obtain adequate mineral elements. In addition, the results of the interaction between dietary energy level in late gestation and time postpartum showed that dietary energy level in late gestation had a relatively significant effect on the mineral content of postpartum colostrum.

There are few reports on the effect of dietary energy levels in late gestation on colostrum, macronutrient milk, and plasma minerals in the postpartum period of jennies, and the exact results need to be further explored. The present results showed that Ca, P, K, Mg, Fe, Cu, Mn, Mo, Se, Zn, and Co in milk were significantly positively correlated with their plasma levels, which showed that the transport of some minerals in milk was passed from plasma to breast milk through passive transport. However, this transport mechanism is determined by the plasma mineral content, so the physical and nutritional status of the dam must be taken into account to ensure the growth and development of the foals. Some of the minerals are obtained through the active transport of the mammary epithelial cells themselves, which leads to the aggregation of certain minerals in the milk to ensure the growth and development of the foal. However, the link between the active transport mechanisms of mammary epithelial cells themselves and minerals needs to be investigated.

More studies have been conducted on the basic components of jennies’ milk, but reports on macronutrients and trace elements in jennies’ milk are limited due to various constraints such as geographical differences, population size, and sample collection methods [30,31,32]. In the present experiments, it was found that jennies’ milk had relatively high contents of Ca and P, which were in the ranges of 888.89–1351.11 mg/L and 648–1218.31 mg/L, respectively, comparable to those of horse milk (500–1300 mg/L, 200–1200 mg/L) [33,34,35], which are 4.2 and 6.1 times greater than the Ca and P in human milk (230–300 mg/L and 130–170 mg/L) [36,37,38], respectively, and about one time lower than the Ca and P in cows’ milk (1120–1230 mg/L, 590–1190 mg/L) [39]. Ca/P was balanced at about 1.2, which is slightly lower than in horse milk (1.3), human milk (1.8), and cow milk (1.3). The contents of the trace elements Fe, Zn, and Mn in jennies’ milk (0.43–2.64 mg/L, 1.23–3.19 mg/L, and 0.0036 mg/L) were similar to those of human milk (0.72 mg/L, 1.00–3.00 mg/L, and 0.0030–0.0060 mg/L), whereas the contents of Cu and Se (0.20–0.40 mg/L, 0.01–0.02 mg/L) were relatively low [29,37,38]. Overall, jennies’ milk is relatively low in minerals and places less burden on the kidneys when consumed [40]. Ca has been consistently reported to be the major element in jennies’ milk [2,41,42] as well as horses’ milk [26] and is essential for bone mineralization in infants, young children, and the elderly [43]. Zn, Fe, and Cu are key in antioxidants, enzyme cofactors, and immune cell activity [44]. The mineral element contents of jennies’ milk reported so far vary relatively widely. The results from this study provide data support and a theoretical basis for the supply of an appropriate amount of energy to pregnant jennies and the optimal production of jennies’ milk, and for understanding the characteristics of the minerals of jennies’ milk.

## 5. Conclusions

Appropriately increasing dietary energy levels in late gestation increased the concentrations of Ca, P, K, Mg, Cu, Fe, Zn, and Mo in milk, but high dietary energy levels showed the opposite effect. The concentrations of these minerals in jenny milk decreased with the duration of lactation.

## Figures and Tables

**Table 1:** The ingredients and nutrient compositions of the diets (dry-matter basis).

	Late Gestation Diets			Lactation Diet
Items	HE ^(4)^	ME ^(4)^	LE ^(4)^	
Ingredients (kg/d per jenny, as fed)				
Millet straw	46.33	57.37	67.16	44.63
Alfalfa hay	18.53	8.91	2.04	20.64
Corn	20.20	16.09	11.56	17.95
Soybean meal	7.53	7.80	6.93	8.27
Corn gluten meal	0.23	1.89	2.85	2.14
Corn germ meal	0.35	1.45	1.43	1.78
Wheat bran	0.46	1.67	2.45	1.71
Distillers dried grains with solubles	0.46	0.45	2.45	0.53
Extruded full-fat soybean	3.55	2.12	1.08	0.00
Premix ^(1)^	0.63	0.61	0.56	0.63
NaCl	0.47	0.45	0.41	0.46
CaCO_3_	0.42	0.40	0.37	0.41
CaHPO_4_	0.83	0.80	0.73	0.83
Total	100.00	100.00	100.00	100.00
Nutrient level (%)				
DE(MJ/kg) ^(2)^	12.54	12.03	11.39	12.40
CP ^(3)^	13.26	13.21	13.17	14.01
EE ^(3)^	3.34	3.22	3.38	2.82
NDF ^(3)^	52.04	54.28	56.80	52.48
ADF ^(3)^	31.08	31.71	32.49	31.15
Ca ^(3)^	1.10	1.10	1.16	0.37
P ^(3)^	0.36	0.36	0.37	14.01

(1) One kilogram of premix provided the following: Vitamin A, 1,200,000 lU; Vitamin D, 250,000 lU; Vitamin E, 3000 lU; Fe, 4 g: Cu, 1.6 g; Zn, 12 g: Mn, 12 g; I,72 mg: Se, 60 mg; Co, 100 mg. (2) Digestible energy, calculated for horses, according to NRC (2007) [13]. (3) CP: crude protein; EE: ether extract; NDF: neutral detergent fiber; ADF: acid detergent fiber; Ca: calcium; P: phosphorus. (4) HE, ME, and LE refer to high-digestible-energy (12.54 MJ/kg), medium-energy (12.03 MJ/kg), and low-energy (11.39 MJ/kg) diets.

**Table 2:** Effect of dietary energy level during late gestation on postpartum macronutrient content in plasma of jennies (mg/L).

Item		DE			PT						SEM	*p*_-DE_	*p*_-PT_
	Distribution Range	HE	ME	LE	0 h	24 h	48 h	5 d	7 d	14 d			
Ca	91.85–125.37	101.05 ^C^	110.99 ^A^	107.74 ^B^	125.37 ^A^	112.93 ^B^	106.87 ^C^	103.73 ^D^	98.81 ^E^	91.85 ^F^	1.659	<0.001	<0.001
P	76.59–117.26	89.24 ^C^	100.46 ^A^	95.21 ^B^	117.26 ^A^	104.59 ^B^	97.35 ^C^	91.78 ^D^	82.25 ^E^	76.59 ^F^	2.637	<0.001	<0.001
K	103.23–134.49	115.02 ^B^	120.43 ^A^	117.34 ^B^	134.49 ^A^	128.09 ^B^	121.92 ^C^	112.15 ^D^	105.70 ^E^	103.23 ^E^	2.333	0.001	<0.001
Mg	23.77–29.36	25.74 ^A^	26.60 ^A^	25.95 ^A^	29.36 ^A^	27.39 ^B^	25.90 ^C^	25.49 ^C^	24.67 ^C,D^	23.77 ^D^	0.863	0.207	<0.001

A–F: means with different superscripts within a column differ significantly (*p* < 0.05). DE: dietary digestible energy levels; PT: postpartum time (days after foaling) (*p* < 0.05). HE, ME, and LE refer to high-digestible-energy (12.54 MJ/kg), medium-energy (12.03 MJ/kg), and low-energy (11.39 MJ/kg) diets.

**Table 3:** Effect of interaction between dietary energy level in late gestation and postpartum time on macronutrient content in plasma of jennies (mg/L).

DE	HE						ME						LE						SEM	*p*-Value
PT	0 h	24 h	48 h	5 d	7 d	14 d	0 h	24 h	48 h	5 d	7 d	14 d	0 h	24 h	48 h	5 d	7 d	14 d		
Ca	120.89 ^b^	106.41 ^f^	99.34 ^g^	97.66 ^g,h^	93.61 ^h,i^	88.42 ^j^	128.20 ^a^	117.96 ^b,c^	112.04 ^d,e^	107.09 ^f^	105.25 ^f^	95.40 ^g–i^	127.01 ^a^	114.41 ^c,d^	109.23 ^e,f^	106.46 ^f^	97.57 ^g,h^	91.74 ^i,j^	1.659	<0.001
P	107.91 ^b–d^	100.13 ^d–f^	90.58 ^g–i^	87.92 ^h,i^	77.83 ^j,k^	71.07 ^j^	128.51 ^a^	108.87 ^b,c^	103.63 ^c–f^	95.99 ^f,g^	85.98 ^h–j^	79.75 ^j^	115.36 ^b^	104.76 ^c–e^	97.83 ^e–g^	91.42 ^g,h^	82.95 ^i,j^	78.96 ^j^	2.637	<0.001
K	131.20 ^a–c^	125.57 ^c–e^	118.69 ^e^	108.37 ^f^	103.91 ^f^	102.35 ^f^	137.99 ^a^	130.60 ^b–d^	123.70 ^d,e^	118.33 ^e^	108.29 ^f^	103.69 ^f^	134.27 ^a,b^	128.08 ^b–d^	123.40 ^d,e,^	109.74 ^f^	104.89 ^f^	103.64 ^f^	2.333	<0.001
Mg	28.61 ^a–c^	26.35 ^c–e^	25.74 ^d–f^	25.61 ^d–f^	24.73 ^d–f^	23.43 ^f^	30.41 ^a^	28.55 ^a–c^	26.02 ^c–f^	25.45 ^d–f^	24.87 ^d–f^	24.28 ^e,f^	29.08 ^a,b^	27.27 ^b–d^	25.94 ^c–f^	25.41 ^d–f^	24.41 ^d–f^	23.60 ^e,f^	0.863	<0.001

a–j: means with different superscripts within a column indicate significant differences in the interaction among DE and PT (*p* < 0.05). DE: dietary digestible energy levels; PT: postpartum time (days after foaling) (*p* < 0.05). HE, ME, and LE refer to high-digestible-energy (12.54 MJ/kg), medium-energy (12.03 MJ/kg), and low-energy (11.39 MJ/kg) diets.

**Table 4:** Effect of dietary energy level during late gestation on postpartum trace element content in plasma of jennies (µg/100 mL).

Item		DE			PT						SEM	*p*_-DE_	*p*_-PT_
	Distribution Range	HE	ME	LE	0 h	24 h	48 h	5 d	7 d	14 d			
Fe	774.59–1173.55	946.65 ^B^	1009.38 ^A^	962.35 ^B^	1173.55 ^A^	1095.36 ^B^	1020.31 ^C^	935.75 ^D^	837.23 ^E^	774.59 ^F^	29.742	0.001	<0.001
Cu	73.48–94.61	84.31 ^A^	87.45 ^A^	84.81 ^A^	94.61 ^A^	92.27 ^A^	88.37 ^AB^	83.87 ^BC^	80.54 ^C^	73.48 ^D^	3.823	0.316	<0.001
Mn	10.72–18.55	14.34 ^B^	15.37 ^A^	14.84 ^AB^	18.55 ^A^	17.30 ^B^	15.73 ^C^	14.51 ^D^	12.30 ^E^	10.72 ^F^	0.503	0.003	<0.001
Zn	100.85–256.97	157.23 ^C^	180.32 ^A^	170.12 ^B^	256.97 ^A^	187.09 ^B^	181.16 ^B^	170.10 ^C^	119.17 ^D^	100.85 ^E^	4.525	<0.001	<0.001
Se	12.38–20.77	16.66 ^B^	17.74 ^A^	17.56 ^A^	20.77 ^A^	19.54 ^B^	18.87 ^B^	17.89 ^C^	14.46 ^D^	12.38 ^E^	0.527	0.001	<0.001
Mo	3.43–5.49	4.34 ^B^	4.92 ^A^	4.73 ^A^	5.49 ^A^	5.39 ^A^	4.97 ^B^	4.51 ^C^	4.21 ^C^	3.43 ^D^	0.235	<0.001	<0.001
Co	0.72–1.38	0.96 ^B^	1.08 ^A^	1.04 ^A^	1.38 ^A^	1.26 ^B^	1.09 ^C^	0.90 ^D^	0.81 ^E^	0.72 ^E^	0.057	0.001	<0.001

A–F: means with different superscripts within a column differ significantly (*p* < 0.05). DE: dietary digestible energy levels; PT: postpartum time (days after foaling) (*p* < 0.05). HE, ME, and LE refer to high-digestible-energy (12.54 MJ/kg), medium-energy (12.03 MJ/kg), and low-energy (11.39 MJ/kg) diets.

**Table 5:** Effect of interaction between dietary energy level in late gestation and postpartum time on trace element content in plasma of jennies (µg/100 mL).

DE	HE						ME						LE						SEM	*p*-Value
PT	0 h	24 h	48 h	5 d	7 d	14 d	0 h	24 h	48 h	5 d	7 d	14 d	0 h	24 h	48 h	5 d	7 d	14 d		
Fe	1121.03 ^b,c^	1080.32 ^b–d^	994.97 ^d–f^	892.33 ^g,h^	829.53 ^h–j^	761.73 ^j^	1235.14 ^a^	1113.10 ^b,c^	1066.07 ^c–e^	981.31 ^e,f^	873.71 ^h–j^	786.97 ^i,j^	1164.47 ^a,b^	1092.66 ^b,c^	999.89 ^d–f^	933.60 ^f,g^	808.43 ^h–j^	775.07 ^j^	29.742	<0.001
Cu	93.56 ^a,b^	91.30 ^a–c^	88.02 ^a–d^	82.14 ^b–e^	80.08 ^c–e^	70.79 ^e^	96.22 ^a^	92.87 ^a–c^	88.85 ^a–d^	86.90 ^a–d^	81.36 ^b–e^	78.48 ^d,e^	94.06 ^a,b^	92.65 ^a–c^	88.23 ^a–c,d^	82.57 ^b–d,e^	80.19 ^c–e^	71.17 ^e^	3.823	<0.001
Mn	18.29 ^a,b^	16.87 ^b–d^	15.40 ^d,e^	13.77 ^f^	11.65 ^h^	10.10 ^i^	18.79 ^a^	17.58 ^a–c^	16.22 ^c,d^	15.34 ^d,e^	13.15 ^f,g^	11.13 ^h,i^	18.57 ^a^	17.47 ^a–c^	15.56 ^d,e^	14.42 ^e,f^	12.10 ^g,h^	10.9 ^h,i^	0.503	<0.001
Zn	213.75 ^c^	185.82 ^d^	174.77 ^d–f^	165.99 ^f^	105.12 ^h,i^	97.91 ^i^	290.05 ^a^	188.08 ^d^	186.19 ^d^	174.62 ^d–f^	139.96 ^g^	103.03 ^h,i^	267.11 ^b^	187.37 ^d^	182.52 ^d,e^	169.70 ^e,f^	112.43 ^h^	101.60 ^h,i^	4.525	<0.001
Se	20.21 ^a,b^	19.23 ^b–d^	18.45 ^c,d^	17.71 ^d^	13.65 ^e,f^	10.71 ^g^	21.11 ^a^	19.71 ^a–c^	19.16 ^b–d^	18.07 ^c,d^	14.95 ^e^	13.42 ^e,f^	21.00 ^a^	19.68 ^a–c^	18.99 ^b–d^	17.88 ^d^	14.77 ^e^	13.02 ^f^	0.527	<0.001
Mo	4.71 ^b–d^	5.34 ^a–c^	4.73 ^b–d^	4.46 ^d–f^	3.95 ^e–g^	2.88 ^h^	5.90 ^a^	5.43 ^a,b^	5.30 ^a–c^	4.66 ^c–e^	4.38 ^d–f^	3.88 ^f,g^	5.87 ^a^	5.41 ^a–c^	4.89 ^b–d^	4.41 ^d–f^	4.29 ^d–f^	3.53 ^g^	0.235	<0.001
Co	1.35 ^a,b^	1.19 ^b–d^	1.04 ^d,e^	0.84 ^f,g^	0.80 ^g^	0.54 ^h^	1.43 ^a^	1.31 ^a,b^	1.13 ^c–e^	1.00 ^e,f^	0.81 ^g^	0.80 ^g^	1.38 ^a^	1.29 ^a–c^	1.10 ^d,e^	0.86 ^f,g^	0.81 ^g^	0.80 ^g^	0.057	<0.001

a–j: means with different superscripts within a column indicate significant differences in the interaction between DE and PT (*p* < 0.05). DE: dietary digestible energy levels; PT: postpartum time (days after foaling) (*p <* 0.05). HE, ME, and LE refer to high-digestible-energy (12.54 MJ/kg), medium-energy (12.03 MJ/kg), and low-energy (11.39 MJ/kg) diets.

**Table 6:** Effect of dietary energy level during late gestation on postpartum macronutrient content in milk of jennies (mg/L).

Item	DE			PT						SEM	*p*_-DE_	*p*_-PT_
	HE	ME	LE	0 h	24 h	48 h	5 d	7 d	14 d			
Ca	1079.43 ^C^	1172.98 ^A^	1110.8 ^B^	1351.11 ^A^	1232.54 ^B^	1137.31 ^C^	1072.88 ^D^	1043.70 ^D^	888.89 ^E^	21.889	<0.001	<0.001
P	904.13 ^C^	1006.44 ^A^	949.62 ^B^	1218.31 ^A^	1114.91 ^B^	1032.65 ^C^	934.50 ^D^	771.57 ^E^	648.43 ^F^	25.168	<0.001	<0.001
K	774.50 ^B^	931.06 ^A^	776.97 ^B^	1539.78 ^A^	1278.12 ^B^	757.32 ^C^	564.94 ^D^	465.73 ^E^	359.17 ^F^	15.947	<0.001	<0.001
Mg	141.93 ^C^	186.97 ^A^	154.71 ^B^	472.26 ^A^	149.60 ^B^	106.74 ^C^	93.79 ^C^	78.99 ^D^	65.86 ^D^	8.336	<0.001	<0.001

A–F: means with different superscripts within a column differ significantly (*p* < 0.05). DE: dietary digestible energy levels; PT: postpartum time (days after foaling) (*p* < 0.05). HE, ME, and LE refer to high-digestible-energy (12.54 MJ/kg), medium-energy (12.03 MJ/kg), and low-energy (11.39 MJ/kg) diets.

**Table 7:** Effect of interaction between dietary energy level in late gestation and postpartum time on macronutrient content in milk of jennies (mg/L).

DE	HE						ME						LE						SEM	*p*-Value
PT	0 h	24 h	48 h	5 d	7 d	14 d	0 h	24 h	48 h	5 d	7 d	14 d	0 h	24 h	48 h	5 d	7 d	14 d		
Ca	1279.13 ^c^	1212.53 ^d^	1112.27 ^e^	1053.34 ^e–g^	1037.07 ^f,g^	782.19 ^i^	1426.13 ^a^	1247.53 ^c,d^	1185.93 ^d^	1102.77 ^e,f^	1051.62 ^e–g^	1023.86 ^g^	1347.99 ^b^	1237.54 ^c,d^	1113.73 ^e^	1062.52 ^e–g^	1042.43 ^f,g^	860.63 ^h^	21.889	<0.001
P	1104.13 ^b–d^	1084.2 ^c–e^	1015.37 ^e,f^	884.00 ^g^	710.16 ^i^	626.93 ^j^	1373.60 ^a^	1143.20 ^b,c^	1042.40 ^d–f^	1002.53 ^f^	809.34 ^h^	667.55 ^i,j^	1177.20 ^b^	1117.33 ^b–d^	1040.19 ^d–f^	916.97 ^g^	795.22 ^h^	650.80 ^i,j^	25.168	<0.001
K	1484.97 ^b^	1210.91 ^d^	621.07 ^f^	542.10 ^g^	436.29 ^h^	351.66 ^i^	1648.44 ^a^	1422.84 ^c^	1021.68 ^e^	600.23 ^f^	517.44 ^g^	375.73 ^i^	1485.92 ^b^	1200.61 ^d^	629.22 ^f^	552.48 ^g^	443.47 ^h^	350.11 ^i^	15.947	<0.001
Mg	391.80 ^c^	134.82 ^e^	102.97 ^f^	87.93 ^f–h^	76.04 ^g–i^	58.04 ^i^	600.53 ^a^	159.71 ^d^	109.10 ^f^	96.87 ^f,g^	84.43 ^f–i^	71.20 ^g–i^	424.45 ^b^	154.27 ^d,e^	108.15 ^f^	96.57 ^f,g^	76.49 ^g–i^	68.34 ^h,i^	8.336	<0.001

a–i: means with different superscripts within a column indicate significant differences in the interaction between DE and PT (*p* < 0.05). DE: dietary digestible energy levels; PT: postpartum time (days after foaling) (*p* < 0.05). HE, ME, and LE refer to high-digestible-energy (12.54 MJ/kg), medium-energy (12.03 MJ/kg), and low-energy (11.39 MJ/kg) diets.

**Table 8:** Effect of dietary energy level in late gestation and postpartum time on trace element content in milk of jennies (ug/100 mL).

Item	DE			PT						SEM	*p*_-DE_	*p*_-PT_
	HE	ME	LE	0 h	24 h	48 h	5 d	7 d	14 d			
Fe	188.88 ^B^	207.50 ^A^	189.93 ^B^	278.47 ^A^	232.04 ^B^	201.24 ^C^	175.29 ^D^	156.87 ^E^	128.71 ^F^	6.221	<0.001	<0.001
Cu	13.20 ^C^	15.11 ^A^	14.07 ^B^	22.11 ^A^	16.22 ^B^	14.47 ^C^	13.37 ^D^	10.32 ^E^	8.26 ^F^	0.644	<0.001	<0.001
Mn	2.61 ^B^	3.13 ^A^	3.06 ^A^	4.71 ^A^	3.51 ^B^	3.06 ^C^	2.61 ^D^	2.16 ^E^	1.58 ^F^	0.103	<0.001	<0.001
Zn	146.33 ^C^	177.51 ^A^	155.96 ^B^	270.49 ^A^	199.35 ^B^	164.45 ^C^	141.48 ^D^	99.26 ^E^	84.57 ^F^	3.845	<0.001	<0.001
Se	0.91 ^B^	1.30 ^A^	1.27 ^A^	2.44 ^A^	1.53 ^B^	1.16 ^C^	0.79 ^D^	0.63 ^E^	0.40 ^F^	0.055	<0.001	<0.001
Mo	0.72 ^C^	0.94 ^A^	0.91 ^B^	1.76 ^A^	1.28 ^B^	0.72 ^C^	0.58 ^D^	0.48 ^E^	0.33 ^F^	0.019	<0.001	<0.001
Co	0.18 ^B^	0.21 ^A^	0.20 ^A^	0.32 ^A^	0.21 ^B^	0.19 ^C^	0.18 ^D^	0.15 ^E^	0.13 ^F^	0.012	<0.001	<0.001

A–F: means with different superscripts within a column differ significantly (*p* < 0.05). DE: dietary digestible energy levels; PT: postpartum time (days after foaling) (*p* < 0.05). HE, ME, and LE refer to high-digestible-energy (12.54 MJ/kg), medium-energy (12.03 MJ/kg), and low-energy (11.39 MJ/kg) diets.

**Table 9:** Effect of interaction between dietary energy level in late gestation and postpartum time on trace element content in milk of jennies (µg/100 mL).

DE	HE						ME						LE						SEM	*p*-Value
PT	0 h	24 h	48 h	5 d	7 d	14 d	0 h	24 h	48 h	5 d	7 d	14 d	0 h	24 h	48 h	5 d	7 d	14 d		
Fe	263.69 ^b^	228.06 ^c,d^	193.53 ^e^	173.98 ^f^	153.89 ^g,h^	120.14 ^j^	306.35 ^a^	239.67 ^c^	216.65 ^e^	177.69 ^e,f^	163.44 ^f,g^	141.21 ^h,i^	265.36 ^b^	228.38 ^c,d^	193.55 ^e^	174.22 ^f^	153.26 ^g,h^	124.78 ^i,j^	6.221	<0.001
Cu	19.47 ^b^	15.22 ^b^	14.10 ^c,d^	13.04 ^d^	10.03 ^e^	7.33 ^f^	23.72 ^a^	17.98 ^b^	14.90 ^c,d^	13.58 ^c,d^	10.64 ^e^	9.83 ^e^	23.13 ^a^	15.47 ^c,d^	14.41 ^c,d^	13.49 ^c,d^	10.27 ^e^	7.61 ^f^	0.644	<0.001
Mn	4.08 ^b^	3.32 ^c,d^	2.77 ^e^	2.41 ^fg^	1.99 ^h,i^	1.12 ^j^	5.10 ^a^	3.60 ^c^	3.22 ^d^	2.74 ^e^	2.29 ^g,h^	1.85 ^i^	4.93 ^a^	3.60 ^c^	3.19 ^d^	2.69 ^e,f^	2.19 ^g,h^	1.77 ^i^	0.103	<0.001
Zn	217.73 ^c^	193.42 ^d^	161.76 ^e^	136.52 ^g^	89.70 ^i,j^	78.83 ^j^	334.16 ^a^	211.11 ^c^	168.05 ^e^	150.87 ^f^	113.36 ^h^	87.49 ^i,j^	259.59 ^b^	193.52 ^d^	163.53 ^e^	137.05 ^g^	94.71 ^i^	87.38 ^i,j^	3.845	<0.001
Se	1.64 ^b^	1.43 ^c,d^	0.88 ^e^	0.71 ^e–g^	0.56 ^g–i^	0.25 ^i^	2.87 ^a^	1.58 ^b,c^	1.32 ^d^	0.85 ^e^	0.68 ^f,g^	0.50 ^h,i^	2.82 ^a^	1.59 ^b,c^	1.29 ^d^	0.82 ^e,f^	0.64 ^g,h^	0.46 ^i^	0.055	<0.001
Mo	1.55 ^b^	0.89 ^d^	0.64 ^g^	0.55 ^i^	0.42 ^k^	0.30 ^l^	1.88 ^a^	1.48 ^c^	0.79 ^e^	0.61 ^g,h^	0.54 ^i^	0.34 ^l^	1.85 ^a^	1.47 ^c^	0.73 ^f^	0.58 ^h,i^	0.49 ^j^	0.34 ^l^	0.019	<0.001
Co	0.23 ^b^	0.21 ^b,c^	0.18 ^c,d^	0.17 ^d,e^	0.14 ^e–g^	0.12 ^g^	0.38 ^a^	0.21 ^b,c^	0.19 ^c,d^	0.18 ^c–e^	0.16 ^d–f^	0.13 ^f,g^	0.35 ^a^	0.21 ^b,c^	0.19 ^c,d^	0.18 ^c,d^	0.16 ^d–f^	0.13 ^f,g^	0.012	<0.001

a–j: means with different superscripts within a column indicate significant differences in the interaction between DE and PT (*p* < 0.05). DE: dietary digestible energy levels; PT: postpartum time (days after foaling) (*p* < 0.05). HE, ME, and LE refer to high-digestible-energy (12.54 MJ/kg), medium-energy (12.03 MJ/kg), and low-energy (11.39 MJ/kg) diets.

**Table 10:** Correlation between plasma and macronutrients in milk of jennies.

Item	P_1_	K_1_	Mg_1_	Ca_2_	P_2_	K_2_	Mg_2_
Ca_1_	0.835	0.801	0.587	0.799	0.811	0.855	0.772
	<0.001	<0.001	<0.001	<0.001	<0.001	<0.001	<0.001
P_1_		0.796	0.585	0.782	0.824	0.839	0.758
		<0.001	<0.001	<0.001	<0.001	<0.001	<0.001
K_1_			0.506	0.733	0.817	0.834	0.673
			<0.001	<0.001	<0.001	<0.001	<0.001
Mg_1_				0.556	0.552	0.620	0.559
				<0.001	<0.001	<0.001	<0.001
Ca_2_					0.835	0.847	0.737
					<0.001	<0.001	<0.001
P_2_						0.843	0.699
						<0.001	<0.001
K_2_							0.807
							<0.001

Subscript 1: elemental Ca, P, K, and Mg in plasma. Subscript 2: elemental Ca, P, K, and Mg in milk.

**Table 11:** Correlation between plasma and trace elements in milk of jennies.

Item	Cu_1_	Mn_1_	Mo_1_	Se_1_	Zn_1_	Co_1_	Fe_2_	Cu_2_	Mn_2_	Mo_2_	Se_2_	Zn_2_	Co_2_
Fe_1_	0.481	0.765	0.626	0.748	0.792	0.719	0.811	0.786	0.827	0.793	0.783	0.833	0.695
	<0.001	<0.001	<0.001	<0.001	<0.001	<0.001	<0.001	<0.001	<0.001	<0.001	<0.001	<0.001	<0.001
Cu_1_		0.504	0.467	0.503	0.540	0.487	0.534	0.530	0.492	0.497	0.494	0.516	0.385
		<0.001	<0.001	<0.001	<0.001	<0.001	<0.001	<0.001	<0.001	<0.001	<0.001	<0.001	<0.001
Mn_1_			0.707	0.804	0.825	0.745	0.796	0.773	0.811	0.803	0.775	0.829	0.696
			<0.001	<0.001	<0.001	<0.001	<0.001	<0.001	<0.001	<0.001	<0.001	<0.001	<0.001
Mo_1_				0.662	0.668	0.591	0.639	0.638	0.697	0.641	0.672	0.683	0.584
				<0.001	<0.001	<0.001	<0.001	<0.001	<0.001	<0.001	<0.001	<0.001	<0.001
Se_1_					0.785	0.735	0.798	0.766	0.808	0.731	0.741	0.782	0.644
					<0.001	<0.001	<0.001	<0.001	<0.001	<0.001	<0.001	<0.001	<0.001
Zn_1_						0.725	0.881	0.880	0.913	0.861	0.896	0.936	0.840
						<0.001	<0.001	<0.001	<0.001	<0.001	<0.001	<0.001	<0.001
Co_1_							0.759	0.749	0.798	0.796	0.774	0.773	0.652
							<0.001	<0.001	<0.001	<0.001	<0.001	<0.001	<0.001
Fe_2_								0.882	0.901	0.893	0.875	0.912	0.783
								<0.001	<0.001	<0.001	<0.001	<0.001	<0.001
Cu_2_									0.887	0.876	0.881	0.904	0.800
									<0.001	<0.001	<0.001	<0.001	<0.001
Mn_2_										0.913	0.935	0.917	0.842
										<0.001	<0.001	<0.001	<0.001
Mo_2_											0.923	0.915	0.811
											<0.001	<0.001	<0.001
Se_2_												0.928	0.878
												<0.001	<0.001
Zn_2_													0.863
													<0.001

Subscript 1: elemental Fe, Cu, Mn, Zn, Se, Mo, and Co in plasma. Subscript 2: elemental Fe, Cu, Mn, Zn, Se, Mo, and Co in milk.

**Table 12:** Average content and distribution ranges of minerals in milk at different times after parturition of jennies.

Item		Ca (mg/L)	P (mg/L)	K (mg/L)	Mg (mg/L)	Fe (μg/100 mL)	Cu (μg/100 mL)	Mn (μg/100 mL)	Zn (μg/100 mL)	Se (μg/100 mL)	Mo (μg/100 mL)	Co (μg/100 mL)
Average content	0 h–24 h	1291.83	1166.61	1408.95	310.93	255.26	19.17	4.11	234.92	1.99	1.52	0.27
	48 h–7 d	1084.63	912.91	596	93.17	177.8	12.72	2.61	135.06	0.86	0.59	0.17
	14 d	888.89	648.43	359.17	65.86	128.71	8.26	1.58	84.57	0.4	0.33	0.13
	0 h–24 h	1232.54–1351.11	1114.91–1218.31	1278.12–1539.78	149.60–472.26	232.04–278.47	16.22–22.11	3.51–4.71	199.35–270.49	1.53–2.44	1.28–1.76	0.21–0.32
Distribution range	48 h–7 d	1043.70–1137.31	771.57–1032.65	465.73–757.32	78.99–106.74	156.87–201.24	10.32–14.47	2.16–3.06	99.26–164.45	0.63–1.16	0.48–0.72	0.15–0.19
	14 d	782.19–1023.86	626.93–667.55	350.11–375.73	58.04–71.20	120.14–141.21	7.33–9.83	1.12–1.85	78.83–87.49	0.25–0.50	0.30–0.34	0.12–0.13

## Data Availability

The data presented in this study are available on request from the corresponding author.

## References

[1] Valldecabres A., Silva-Del-Río N. (2022). First-milking colostrum mineral concentrations and yields: Comparison to second milking and associations with serum mineral concentrations, parity, and yield in multiparous Jersey cows. J. Dairy Sci..

[2] Fantuz F., Ferraro S., Todini L., Piloni R., Mariani P., Salimei E. (2012). Donkey milk concentration of calcium, phosphorus, potassium, sodium, and magnesium. Int. Dairy J..

[3] Hui F., Yin G.L., Yue Y.X., Zhao Y.L., Guo X.Y., Zhang L., Shi B.L., Yan S.M. (2020). Variation research in serum and milk mineral content of lactating donkeys at different times after parturition. Feed Res..

[4] Hu P., Yang H., Lv B., Zhao D., Zhu W. (2018). Dynamic changes of fatty acids and minerals in sow milk during lactation. J. Anim. Physiol. Anim. Nutr..

[5] Long J.F., Guo S.G., Cai L.C., Zang T.Y., Chen W.B., Xie C.Y. (2020). Variation in milk minerals and chemical components corresponding to milking time and lactation day in sows. Biol. Rhythm. Res..

[6] Mashek D.G., Beede D.K. (2001). Peripartum responses of dairy cows fed energy-dense diets for 3 or 6 weeks prepartum. J. Dairy Sci..

[7] Revell D.K., Williams I.H., Mullan B.P., Ranford J.K., Smits R.J. (1998). Body composition at farrowing and nutrition during lactation affect the performance of primiparous sows: I. Voluntary feed intake, weight loss, and plasma metabolites. J. Anim. Sci..

[8] Messias D.B.C.M., Mounier A.M., Prunier A. (1998). Does feed restriction mimic the effects of increased ambient temperature in lactating sows?. J. Anim. Sci..

[9] Fang L.H., Jin Y.H., Jeong J.H., Hong J.S., Kim Y.Y. (2019). Effects of dietary energy and protein levels on reproductive performance in gestating sows and growth of their progeny. J. Anim. Sci. Technol..

[10] Heo S., Yang Y.X., Jin Z., Park M.S., Yang B.K., Chae B.J. (2008). hormones, milk compositions and reproductive performance in primiparous sows. Can. J. Anim. Sci..

[11] Che L.Q., Hu L., Wu C., Xu Q., Wu D. (2019). Effects of increased energy and amino acid intake in late gestation on reproductive performance, milk composition, metabolic, and redox status of sows. J. Anim. Sci..

[12] Guo Y.M., Yin G.L., Hui F., Guo X.Y., Shi B.L., Zhao Y.L., Yan S.M. (2024). Effects of dietary energy level on antioxidant capability, immune function and rectal microbiota in late gestation donkeys. Front. Microbiol..

[13] NRC (2007). Nutrient Requirements of Horses.

[14] Kira C.S., Maio F.D., Maihara V.A. (2004). Comparison of partial digestion procedures for determination of Ca, Cr, Cu, Fe, K, Mg, Mn, Na, P, and Zn in milk by inductively coupled plasma-optical emission spectrometry. J. AOAC Int..

[15] Fantuz F., Ferraro S., Todini L., Piloni R., Mariani P., Malissiova E., Salimei E. (2015). Minor and potentially toxic trace elements in milk and blood serum of dairy donkeys. J. Dairy Sci..

[16] Yang Y.X., Heo S., Jin Z., Yun J., Shinde P., Choi J., Yang B., Chae B. (2008). Effects of dietary energy and lysine intake during late gestation and lactation on blood metabolites, hormones, milk composition and reproductive performance in multiparous sows. Arch. Anim. Nutr..

[17] Hare K.S., Emily C., Wood K.M., Steele M.A. (2021). 528 Late-Breaking: Late Gestation Metabolizable Energy Intake Increases Colostrum Yield and Alters Colostrum Composition in Beef Cattle. J. Anim. Sci..

[18] Zhou A., Liu G., Jiang X. (2023). Characteristic of the components and the metabolism mechanism of goat colostrum: A review. Anim. Biotechnol..

[19] Nguyen P.H., Sanghvi T., Kim S.S., Tran L.M., Menon P. (2017). Factors influencing maternal nutrition practices in a large scale maternal, newborn, and child health program in Bangladesh. PLoS ONE.

[20] Blum J.W., Hammon H. (2000). Colostrum effects on the gastrointestinal tract, and on nutritional, endocrine and metabolic parameters in neonatal calves. Livest. Prod. Sci..

[21] Blättler U., Hammon H.M., Morel C., Philipona C., Rauprich A., Romé V., Le Huërou-Luron I., Guilloteau P., Blum J.W. (2001). Feeding colostrum, its composition and feeding duration variably modify proliferation and morphology of the intestine and digestive enzyme activities of neonatal calves. J. Nutr..

[22] Chavatte-Palmer P., Duvaux-Ponter C., Clement F. (2001). Passive transfer of immunity in horses. Pferdeheilkunde-Equine Med..

[23] Xing Y.H. (2004). Nutritional value of colostrum and utilisation techniques. China Feed..

[24] Dong L.L., Dan F.Z., Wen Q.J., Jan M.W., Xang W. (2001). Determination of Conventional Components and Minerals in Bovine Colostrum. Xinjiang Agric. Sci..

[25] Csapó-Kiss Z., Stefler J., Martin T.G., Makray S., Csapó J. (1995). Composition of mares’ colostrum and milk. protein content, amino acid composition, and contents of macro and micro-elements. Int. Dairy J..

[26] Summer A., Sabbioni A., Formaggioni P., Mariani P. (2004). Trend in ash and mineral element content of milk from haflinger nursing mares throughout six lactation months. Livest. Prod. Sci..

[27] Liao Q.C., Li Z., Han Y.W., Deng L. (2021). Comparative analysis of serum mineral and biochemical parameter profiles between late pregnant and early lactating jennies. J. Equine Vet. Sci..

[28] Shawaf T., Almathen F., Meligy A., El-Deeb W., Al–Bulushi S. (2017). Biochemical analysis of some serum trace elements in donkeys and horses in Eastern region of Kingdom of Saudi Arabia. Vet. World.

[29] Fantuz F., Ferraro S., Todini L., Mariani P., Piloni R., Salimei E. (2013). Essential trace elements in milk and blood serum of lactating donkeys as affected by lactation stage and dietary supplementation with trace elements. Animal.

[30] Martini M., Altomonte I., Tricò D., Lapenta R.M., Salari F. (2021). Current knowledge on functionality and potential Therapeutic uses of Donkey milk. Animals.

[31] Fantuz F., Stefano F., Todini L., Cimarelli L., Salimei E. (2020). Distribution of calcium, phosphorus, sulfur, magnesium, potassium, and sodium in major fractions of donkey milk. J. Dairy Sci..

[32] Malacarne M., Criscione A., Franceschi P., Bordonaro S., Formaggioni P., Marletta D., Summer A. (2019). New insights into chemical and mineral composition of donkey milk throughout nine months of lactation. Animals.

[33] Csapó J., Stefler J., Martin T.G., Makray S., Csapó–Kiss Z. (1995). Composition of mares’ colostrum and milk. fat content, fatty acid composition, and vitamin content. Int. Dairy. J..

[34] Doreau M., Martin-Rosset W. (2011). Animals That Produce Dairy Foods: Horse. Encyclopedia of Dairy Sciences.

[35] Marconi E., Panfili G. (1998). Chemical composition and nutritional properties of commercial products of mare milk powder. J. Food Compos. Anal..

[36] Salimei E., Fantuz F. (2012). Equid milk for human consumption. Int. Dairy J..

[37] Aspri M., Economou N., Papademas P. (2016). Donkey milk: An overview on functionality, technology, and future prospects. Food Rev. Int..

[38] Altomonte I., Salari F., Licitra R., Martini M. (2018). Donkey and human milk: Insights into their compositional similarities. Int. Dairy J..

[39] Claeys W.L., Verraes C., Cardoen S., De Block J., Huyghebaert A., Raes K., Dewettinck K., Herman L. (2014). Consumption of raw or heated milk from different species: An evaluation of the nutritional and potential health benefits. Food Control..

[40] Brown K.H., Agostoni C., Brunser O. (2007). Breastfeeding and complementary feeding of children up to 2 years of age. Nestle Nutr. Workshop.

[41] Massouras T., Triantaphyllopoulos K.A., Theodossiou I. (2017). Chemical composition, protein fraction, and fatty acid profile of donkey milk during lactation. Int. Dairy J..

[42] Salimei E., Fantuz F., Coppola R., Chiofalo B., Polidori P., Varisco G. (2004). Composition and characteristics of ass’s milk. Anim. Res..

[43] Mansueto P., Iacono G., Taormina G., Seidita A., Carroccio A. (2013). Ass’s Milk in Allergy to Cow’s Milk Protein: A Review. Acta. Medica. Mediterr..

[44] Goff J.P. (2018). Invited review: Mineral absorption mechanisms, mineral interactions that affect acid-base and antioxidant status, and diet considerations to improve mineral status. J. Dairy Sci..

